# Vandergrift’s United States Tariff for 1894

**Published:** 1894-07

**Authors:** 


VANDEGRIFT'S UNITED STATES TARIFF FOR 1894. Published by F. B. Vandegrift & Co., Custom-House Brokers, Tariff Experts, and General Forwarders, 50 South Fourth Street, Philadelphia, Pa., and 27 William Street, New York City. Price, $1.50.

This book will be published seventy-two hours after the President signs the same and will be the recognized authority.

As the publisher well says: "Never before has a book of this kind been compiled, and its want has long since been felt by all classes of manufacturers and dealers having relations with foreign countries."

It is especially compiled for manufacturers and dealers and it gives the list of articles classified under the proper headings for ready reference, together with the rate of duty, paragraph of the law and decisions of the courts; also the allowance of wastage, showing duty to be returned on manufactured articles exported with benefit of drawback; the values of all foreign coins; a condensed express tariff and other useful matters in connection with the Customs service.

So important do we hold this book to be, that it is a matter of surprise nothing of the kind was ever produced before. Not alone to the business man is such a work useful, but it is valuable as well to the voter, who, interested in the great controversies upon the tariff, would be glad to have the detailed schedule before him that he might verify the claims make by the opposing sides in the arguments.

